# Different injection speeds produce distinct temporal dynamics of optic nerve sheath diameter increase during caudal block in children: a prospective randomized trial

**DOI:** 10.3389/fped.2026.1855287

**Published:** 2026-06-09

**Authors:** Wenshuang Yang, Ding Han, Siyuan Xie, Shiya Zou, Ya Ma, Guimin Huang, Shoudong Pan

**Affiliations:** 1 Anesthesiology, Capital Center for Children's Health, Capital Medical University, Capital Institute of Pediatrics, Beijing, China; 2 Ultrasound Department, Capital Center for Children's Health, Capital Medical University, Capital Institute of Pediatrics, Beijing, China; 3 Big Data Center, Capital Center for Children's Health, Capital Medical University, Capital Institute of Pediatrics, Beijing, China

**Keywords:** caudal block, injection speed, optic nerve sheath diameter, pediatric, safety, temporal dynamics

## Abstract

**Background:**

Caudal block increases optic nerve sheath diameter (ONSD), but its temporal dynamics vary considerably across centers. Injection speed is one of the unstandardized factors in caudal block, and whether it influences ONSD dynamics remains unclear.

**Purpose:**

We therefore evaluated the effect of injection speed on ONSD changes during caudal block in children.

**Design:**

A prospective, randomized, observer-blinded trial.

**Methods:**

This study enrolled children aged 1–3 years undergoing perineal surgery. Participants received a caudal block with 0.2% ropivacaine (0.5 mL/kg) at either 1 mL/s (high-speed) or 0.1 mL/s (low-speed). ONSD was measured at baseline, immediately after injection, and at 10, 20 and 40 min time points. The primary outcome was ONSD change over time. Linear mixed-effects models were used for analysis.

**Results:**

Eighty children were analyzed. At T2, ONSD was significantly higher in the high-speed group (4.73 ± 0.43 mm) than in the low-speed group (4.52 ± 0.35 mm, *P* = 0.027); at T3, ONSD was significantly higher in the low-speed group (4.73 ± 0.57 mm) than in the high-speed group (4.49 ± 0.42 mm, *P* = 0.010). Both groups showed significant ONSD increases from T1 onward (*P* < 0.05), without return to baseline by T4. A significant time-by-group interaction (*P* < 0.001) indicated different temporal patterns. The maximum ONSD value did not differ between groups (4.8 mm, *p* = 0.537). No significant differences were observed in mean arterial pressure, heart rate, or end-tidal carbon dioxide at any time point (*P* > 0.05). No adverse events occurred.

**Conclusion:**

At an injection volume of 0.5 mL/kg, both speeds were well tolerated. Faster injection advances the observed ONSD peak; slower injection produces a more gradual rise and is therefore recommended for routine practice.

**Clinical trial registration:**

https://www.chictr.org.cn, identifier ChiCTR2200057459.

## 1 Introduction

Caudal block is widely regarded as safe in pediatric patients (1, 2). Nevertheless, real-time ultrasonography shows that a single caudal bolus injection increases optic nerve sheath diameter (ONSD), reflecting a transient rise in intracranial pressure (ICP) (3–5). This elevation has not been reported to cause pathological changes in healthy children, but it highlights a potential risk of this procedure on intracranial dynamics (6–8).

If such transient ICP elevation is inevitable, then identifying modifiable factors that influence its time course becomes clinically relevant. Following a single caudal block, ONSD rapidly increases and then slowly declines, correlating positively with volume (3, 4). However, the time course varies considerably across centers (3, 4). Injection speed is an operator-dependent variable that remains unstandardized; few studies have investigated its relationship with ONSD dynamics, and existing reports are scarce and conflicting (9, 10). Whether high speed affects the temporal characteristics of ONSD compared with low speed remains unclear. In children older than 1 year, an ONSD threshold of 5.75 mm indicates intracranial hypertension (11), but it is unknown whether different injection speeds lead to ONSD values approaching or exceeding this threshold.

We therefore hypothesized that injection speed alters the temporal dynamics of ONSD during caudal block in children. In addition, we aimed to assess whether any ONSD values would approach or exceed the 5.75 mm threshold for intracranial hypertension, and to record any related adverse events as safety outcomes.

## 2 Materials and methods

### 2.1 Study design and registration

**Figure 1:**
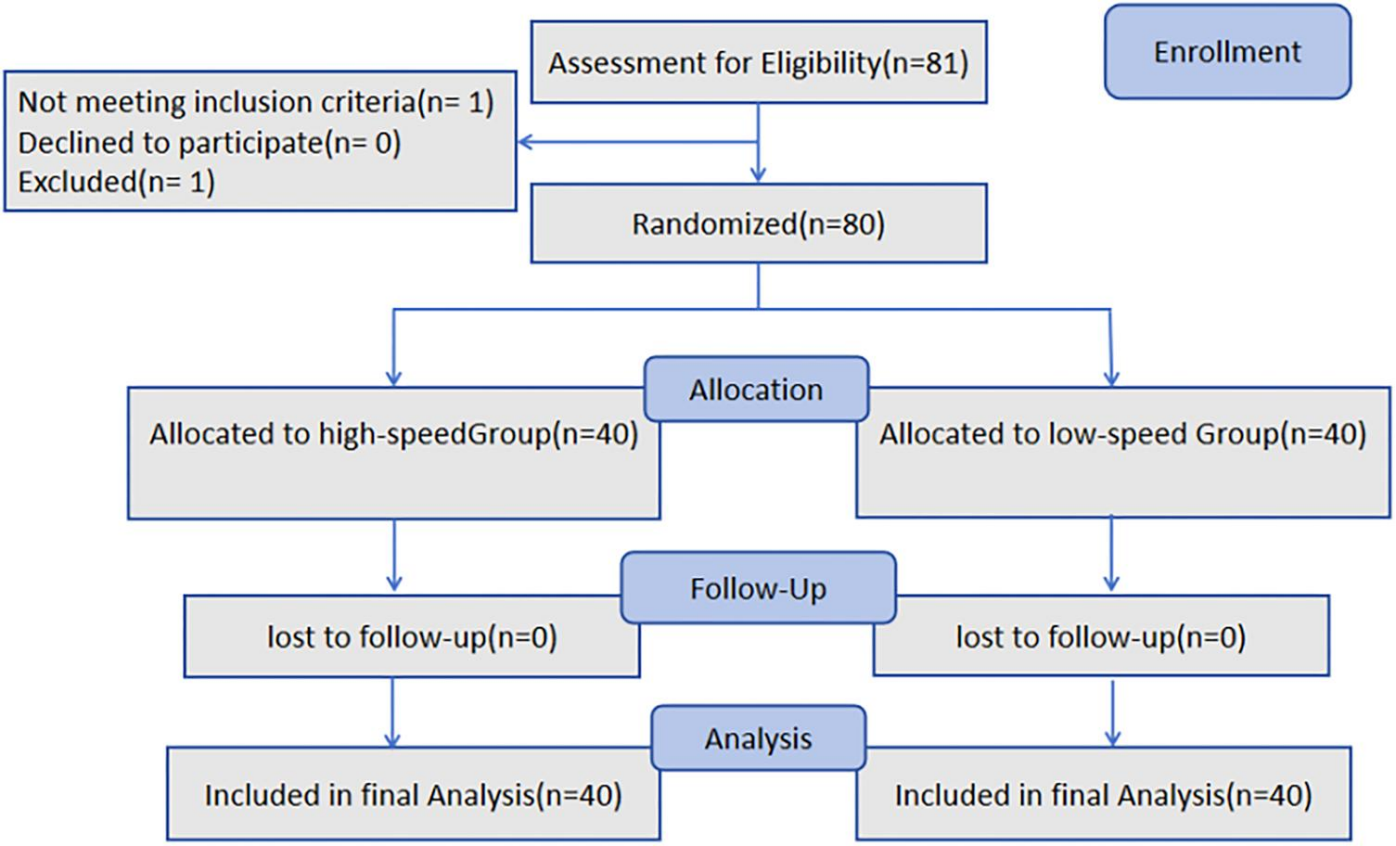
Consolidated standards of reporting trials (CONSORT) flow diagram of participant enrolment, allocation, and follow-up.

This was a prospective, parallel-group, randomized, observer-blinded trial. The study was approved by the Institutional Ethics Committee (SHERLL2022004) and registered with the Chinese Clinical Trials Registry (ChiCTR2200057459). The report adheres to CONSORT guidelines. The trial flow diagram has been submitted in Figure 1. Patient recruitment occurred from March 2022 (first patient enrollment date) to March, 2023 (last patient enrollment date). Before enrollment, the legal guardians of all children were fully informed of the trial protocol and signed a written informed consent.

### 2.2 Participants

Children aged 1–3 years, ASA physical status I–II, scheduled for perineal surgery were eligible. Exclusion criteria were: expected surgical duration <1 h; sacral/coccygeal deformities; ocular pathologies; prior ophthalmic surgery; intracranial hypertension or space-occupying lesions (persistent headache with vomiting, bulging fontanelle in infants, macrocephaly, or a documented history of idiopathic intracranial hypertension, or prior intracranial surgery, such as craniotomy, burr hole, or shunt placement); and history of local anesthetic allergy.

### 2.3 Randomization and blinding

This was a parallel-group, randomized, controlled, single-blind trial. Participants were randomized (1:1) to receive a single-shot caudal block using 0.2% ropivacaine (0.5 mL/kg) administered at either 1 mL/s (high-speed) or 0.1 mL/s (low-speed) using a computer-generated random sequence concealed in opaque envelopes. The anesthesiologist performing the block was unblinded, but the operator measuring ONSD and the data analyst were blinded to group assignment.

### 2.4 Anesthesia, caudal blocks, ONSD measurement and equipment

#### 2.4.1 Anesthesia protocol

Standard intraoperative monitoring included heart rate (HR), non-invasive blood pressure (NIBP), and peripheral oxygen saturation (SpO₂). General anesthesia was induced with intravenous administration of sufentanil (0.2 μg/kg), rocuronium (0.3 mg/kg), propofol (3 mg/kg), and atropine (0.01 mg/kg). After laryngeal mask airway (LMA) insertion, mechanical ventilation was adjusted to maintain end-tidal carbon dioxide (EtCO₂) at 35–45 mmHg. Anesthesia was maintained with continuous infusion of propofol (10 mg/kg/h). Doses of intravenous anesthetics were titrated to maintain blood pressure within ±20% of baseline values. No atropine was administered during the procedure unless the patient's heart rate fell by more than 20% from baseline.

#### 2.4.2 Caudal block procedure

**Figure 2:**
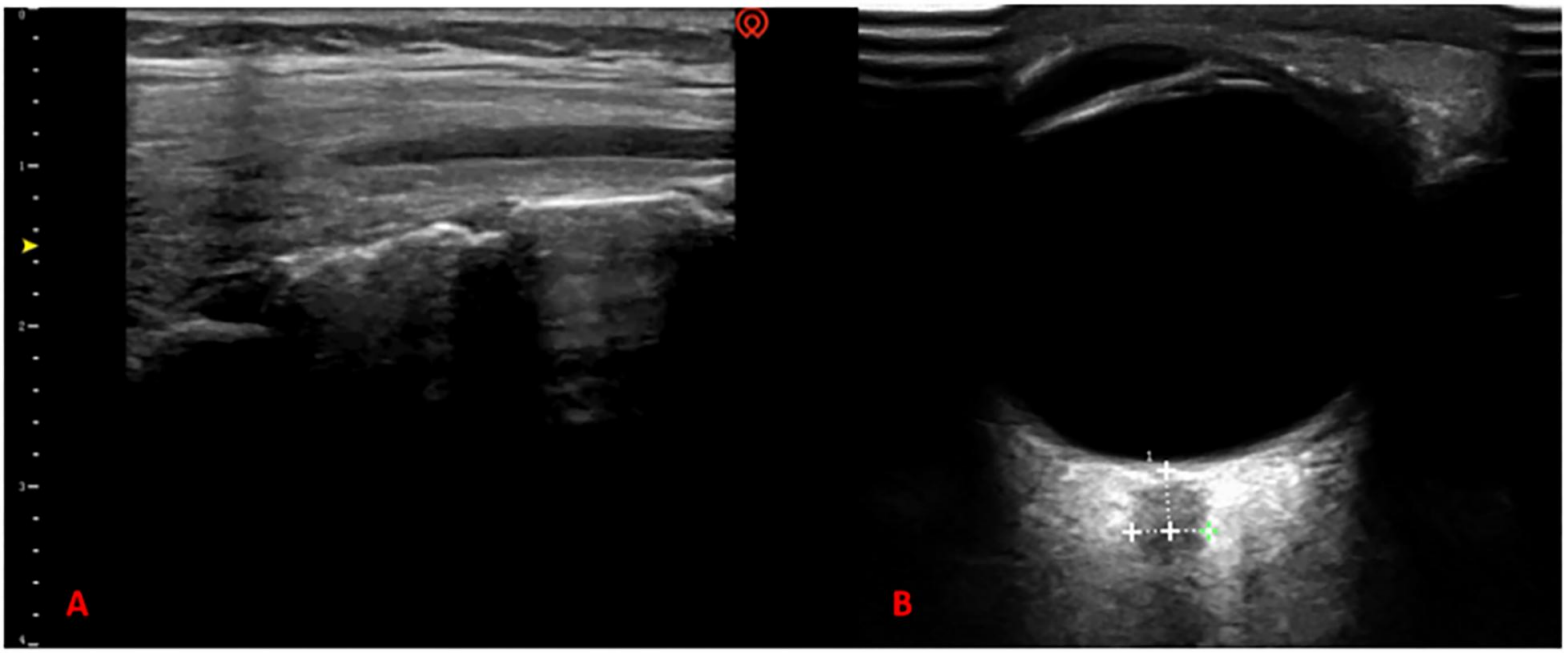
**(A)** the caudal block was administed with visual confirming by ultrasonography. **(B)** Measurement of optic nerve sheath diameter (ONSD) by ultrasonography. 1 vertical axis, 3 mm posterior to the optic nerve head; 2 horizontal axis, the width of ONSD.

After inducing general anesthesia, an angiocatheter was inserted into the sacral canal under ultrasound guidance, and the caudal block was administered using 0.2% ropivacaine with visual confirmation of injectate spread (Figure 2A).

0.2% ropivacaine was prepared in a 20 mL syringe. The syringe was mounted on an infusion pump and connected to the needle hub via infusion pump tubing, which was primed before the caudal block. The caudal block was performed under ultrasound guidance. After successful puncture, the calculated volume of local anesthetic was administered at the predetermined rate according to group assignment. The entire infusion process was conducted under continuous ultrasound visualization to ensure successful block.

#### 2.4.3 ONSD measurement

A single blinded operator performed bilateral ultrasonographic ONSD measurements 3 mm behind the globe using a 7–12 MHz linear probe. The probe was placed gently on closed eyelids in the coronal plane to visualize the optic nerve 3 millimeters (mm) behind the globe. Three measurements were averaged (Figure 2B).

#### 2.4.4 Equipment

Infusion pump: Lead Fluid Syringe Pump YYD02-02 (Baoding Lead Fluid Technology Co., Ltd, China).

Ultrasound equipment: Wisonic-NaviX with 7–12 MHz linear array probe (Wisonic Medical Technology Ltd, Shenzhen, China).

### 2.5 Interventions

Patients were randomly assigned to one of two groups using a computer-generated sequence. The high-speed group (*n* = 40) received 0.2% ropivacaine (0.5 mL/kg) at an injection rate of 1 mL/s, whereas the low-speed group (*n* = 40) received the same volume and concentration at 0.1 mL/s. A 20 mL syringe containing the calculated volume of local anesthetic was mounted on an infusion pump (Lead Fluid Syringe Pump YYD02-02) and connected to the caudal needle hub via primed tubing. Under real-time ultrasound guidance (Wisonic-NaviX, 7–12 MHz linear probe), an angiocatheter was inserted into the sacral canal. After confirming correct needle placement, the local anesthetic was administered at the predetermined rate, and the entire injection was visualized continuously to ensure proper epidural spread. The operator performing ONSD measurements was blinded to group allocation.

### 2.6 Outcome measures

The primary outcome was the change in ONSD over time, measured at five prespecified time points: baseline (T0), immediately after caudal block (T1), 10 min (T2), 20 min (T3), and 40 min (T4) after injection. T0 ONSD was measured after the induction but before caudal block.

Secondary outcomes included hemodynamic and other parameters associated with intracranial pressure, including mean arterial pressure (MAP), heart rate (HR), and end-tidal carbon dioxide (EtCO₂) at each time point.

Adverse events related to possible intracranial hypertension, including postoperative nausea, vomiting, altered mental status, were assessed by a blinded observer continuously during the 30-minute observation period in the post-anesthesia care unit. Any neurological symptom prompted immediate clinical evaluation and, if indicated, neuroimaging.

### 2.7 Sample size

Based on prior data (3, 12), we considered a difference in ONSD > 0.3 mm {10% of mean ONSD in asymptomatic pediatric subjects [mean ONSD 3.08 (SD 0.36) mm]} to be clinically relevant. Considering a significance level of 5% and power of 80%, 31 subjects were required in each group. Based on the precedent set from a prior study (3) on caudal block, we increased the sample size to 40 patients per group.

### 2.8 Statistical analysis

Continuous data are presented as mean ± SD; categorical data as number (%). Comparisons of ONSD over time between groups were performed using linear mixed-effects models (LMMs) with group, time, their interaction, as well as MAP, HR, and EtCO₂ as covariates, as fixed effects and subject as a random effect. Post-hoc pairwise comparisons with Tukey adjustment were conducted when a significant interaction was detected.

MAP, HR, and EtCO₂ over time were compared between groups using the same LMMs (without additional covariates). The highest recorded ONSD value for each patient (defined as the maximum among the five measurements) was compared between groups using an independent t-test (or Mann-Whitney U test if non-normal).

Demographic data were compared using t-tests or chi-square tests, as appropriate. Adverse events were summarized descriptively (number and percentage). A two-sided *P*-value < 0.05 was considered statistically significant. All analyses were performed using RStudio (version 4.3.2).

## 3 Results

### 3.1 Participant ﬂow

Eighty patients who met the inclusion criteria were enrolled and randomized in a 1:1 ratio to the high-speed group (*n* = 40) or the low-speed group (*n* = 40). No patients withdrew from the study or were lost to follow-up. Thus, all 80 patients (all were male) completed the protocol and were included in the final analysis. The flow diagram is shown in Figure 1.

### 3.2 Baseline and intraoperative characteristics

Baseline characteristics are presented in Table 1. The high speed-group and low-speed group were well balanced with respect to age, BMI, and ASA physical status. No statistically significant differences were observed between groups in anesthesia time (76.78 ± 10.74 vs. 77.00 ± 10.55 min; *P* = 0.925) or PACU time (33.7 ± 4.9 vs. 34.4 ± 5.2 min; *P* = 0.553).

**Table 1:** Baseline and patient characteristics.

Variables	high-speed group (*n* = 40)	low-speed group (*n* = 40)	95%CI	*P* value
Age (months)	22.50 ± 7.34	22.75 ± 6.77	(−3.39, −2.89)	0.875
Weight(kg)	11.57 ± 2.09	11.62 ± 1.99	(−0.95, −0.86)	0.917
Height(cm)	84.46 ± 10.94	85.59 ± 18.11	(−7.79, −5.54)	0.738
BMI(kg/m^2^)	16.43 ± 2.59	16.54 ± 3.14	(−1.40, −1.16)	0.856
ASA(I/II)	33/7	32/8	NA	NA
Anesthesia time(min)	76.78 ± 10.74	77.00 ± 10.55	(−4.96, 4.51)	0.925
PACU time(min)	33.70 ± 4.92	34.38 ± 5.21	(−2.93, 1.58)	0.553

Data are presented as mean ± SD.

Interventions: The high-speed group received 0.2% ropivacaine (0.5 mL/kg) at an injection rate of 1 mL/s, and the low-speed group at 0.1 mL/s.

CI, confidence interval; BMI, body mass index; ASA, American Society of Anesthesiologists; PACU, post-anesthesia care unit; NA, not applicable.

### 3.3 Primary outcome

In both groups, ONSD increased significantly from T1 onward compared to T0 (all *P* < 0.05), and had not returned to baseline by T4.

**Figure 3:**
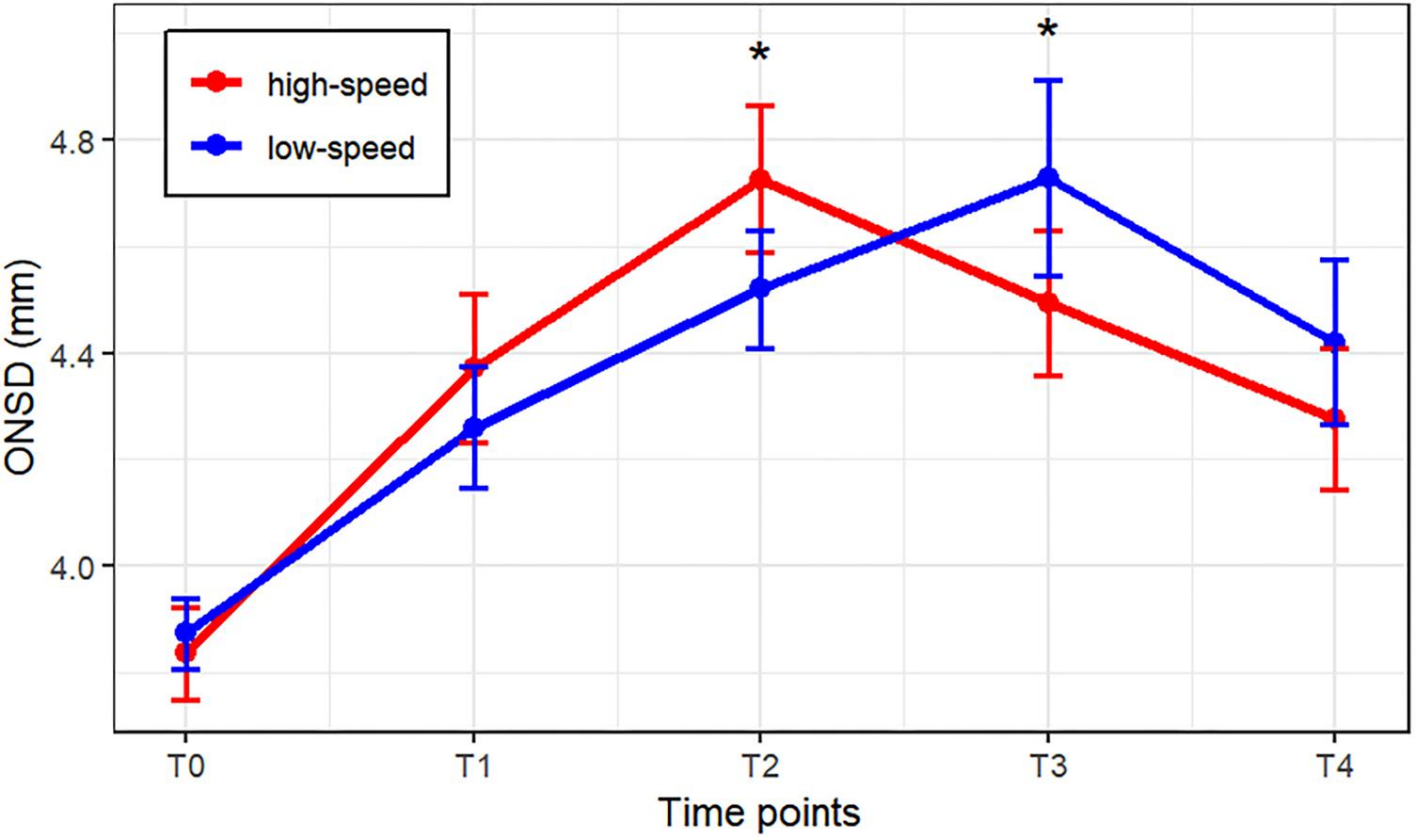
Temporal changes in ONSD in the high-speed and low-speed groups. After adjusting for mean arterial pressure, heart rate, and end-tidal carbon dioxide levels, the linear mixed-effects model revealed a significant time  ×  group interaction (*P* *<* 0.001). *P < 0.05. Interventions: The high-speed group received 0.2% ropivacaine (0.5 mL/kg) at an injection rate of 1 mL/s, and the low-speed group at 0.1 mL/s. ONSD, optical nerve sheath diameter. T0 (after induction,before caudal block),T1(after caudal block), T2 (10 min after caudal block), T3 (20 min after caudal block), T4 (40 min after caudal block).

At T2, ONSD was 4.73 ± 0.43 mm in the high-speed group, and 4.52 ± 0.35 mm in the low-speed group; at T3, ONSD was 4.73 ± 0.57 mm in the low-speed group, and 4.49 ± 0.42 mm in the high-speed group. After adjusting for MAP, HR, and EtCO₂, the linear mixed-effects model revealed a significant time  ×  group interaction (*P* < 0.001). Post-hoc comparisons with Tukey correction showed that ONSD was significantly higher in the high-speed group at T2 (*P* = 0.027) and significantly higher in the low-speed group at T3 (*P* = 0.010) (Table 2, Figure 3).

**Table 2:** Comparison of ONSD at different points of observation between groups.

Times	ONSD of high-speed Group (*n* = 40)	ONSD of low-speed Group (*n* = 40)	95% CI of difference between Groups	Ajusted *P* value
T0	3.84 ± 0.27	3.88 ± 0.21	(−0.013, 0.022)	0.624
T1	4.37 ± 0.43	4.26 ± 0.35	(−0.030, 0.006)	0.205
T2	4.73 ± 0.43	4.52 ± 0.35	(−0.038, −0.002)	0.027*
T3	4.49 ± 0.42	4.73 ± 0.57	(0.006, 0.041)	0.010*
T4	4.28 ± 0.41	4.42 ± 0.49	(−0.003, 0.033)	0.091
Maximum ONSD distribution	1/34/3/2 (T1/T2/T3/T4)	1/11/28/0 (T1/T2/T3/T4)	NA	NA

Data are presented as mean ± SD.

Interventions: The high-speed group received 0.2% ropivacaine (0.5 mL/kg) at an injection rate of 1 mL/s, and the low-speed group at 0.1 mL/s.

CI, confidence interval; ONSD,optic nerve sheath diameter; NA, not applicable.

T0 (after induction,before caudal block), T1 (after caudal block), T2 (10 min after caudal block), T3 (20 min after caudal block), T4 (40 min after caudal block).

The model was adjusted for heart rate, mean arterial pressure, and end-tidal carbon dioxide levels. The presented *P*-values are Tukey-adjusted for multiple comparisons. *P < 0.05.

No significant between-group differences were observed at T0, T1, or T4 (all *P* *>* 0.05).

Based on the five serial measurements, the time point with the highest ONSD value was T1 for 1 patient (2.5%), T2 for 34 (85.0%), T3 for 3 (7.5%), and T4 for 2 (5.0%) in the high-speed group. In the low-speed group, the highest ONSD was observed at T1 in 1 (2.5%), at T2 in 11 (27.5%), at T3 in 28 (70.0%), and at T4 in 0 (0%) (Table 2).

The distribution of the time points at which the maximum recorded ONSD occurred is shown in Table 2. The highest recorded ONSD value did not differ between groups (high-speed: 4.8 ± 0.40 mm; low-speed: 4.8 ± 0.50 mm; *P* = 0.537).

### 3.4 Secondary outcomes

No significant intergroup differences were observed in MAP, HR, or EtCO₂ at any time point (Table 3). No adverse events related to possible intracranial hypertension (including nausea, vomiting, altered mental status) occurred in either group.

**Table 3:** Comparison of HR, MAP, EtCO2 at each time point .

Variables	T0	T1	T2	T3	T4
High-speed HR	122 ± 7	122 ± 9	125 ± 5	124 ± 6	121 ± 6
Low-speed HR	121 ± 6	121 ± 6	125 ± 6	124 ± 5	121 ± 5
*P* Value	0.656	0.318	0.721	0.929	0.721
High-speed MAP	74 ± 3	76 ± 4	74 ± 3	75 ± 3	74 ± 3
Low-speed MAP	73 ± 2	74 ± 3	75 ± 4	74 ± 3	74 ± 3
*P* Value	0.502	0.068	0.479	0.709	0.852
High-speed EtCO_2_	40 ± 4	39 ± 4	39 ± 4	39 ± 3	40 ± 4
Low-speed EtCO_2_	39 ± 3	40 ± 3	38 ± 3	39 ± 3	39 ± 3
*P* Value	0.347	0.221	0.638	0.754	0.065

Data are presented as mean ± SD.

Interventions: The high-speed group received 0.2% ropivacaine (0.5 mL/kg) at an injection rate of 1 mL/s, and the low-speed group at 0.1 mL/s.

CI, confidence interval; MAP, mean arterial pressure; HR, heart rate; and EtCO_2_, end-tidal carbon dioxide.

T0 (after induction,before caudal block), T1 (after caudal block), T2 (10 min after caudal block), T3 (20 min after caudal block), T4 (40 min after caudal block).

The presented *P*-values are Tukey-adjusted for multiple comparisons.

## 4 Discussion

This study revealed that injection speed significantly alters the temporal dynamics of ONSD following caudal block. Faster injection advanced the observed peak ONSD time without changing the maximum value. All ONSD values remained below the 5.75 mm threshold, and no adverse events occurred. Systemic hemodynamics and EtCO_2_ were unaffected by injection speed.

Our findings help reconcile conflicting reports. One study using 1 mL/s vs. 3 mL/s found no difference in ONSD at each time point (9), whereas another with a 16.7-fold faster injection led to an earlier peak, and slower infusion resulted in a higher maximum ONSD (10). A recently published study with a 2-fold speed difference (0.5 vs. 0.25 mL/s) reported a statistically significant but numerically minimal ONSD increase (4.43 ± 0.09 vs. 4.47 ± 0.11 mm, *P* = 0.042) immediately after injection, with no between -group differences at subsequent time points (13). Taken together, these observations raise the possibility of a threshold effect: relatively small speed differences (2- to 3-fold) may be insufficient to produce clear changes in ONSD kinetics, or may only induce minimal, transient effects, whereas a sufficiently large speed difference (≥10-fold, as in our 10-fold study and the 16.7-fold study) appears to generate a discernible temporal separation. However, direct cross study comparisons are limited by differences in patient age, local anesthetic volume, anesthetic regimens, and time point sampling density. Therefore, the existence and precise cutoff of such a threshold remain to be confirmed by future studies specifically designed to isolate the effect of injection speed while controlling for these confounders.

Studies on clinical outcomes have shown that local anesthetic spread is primarily determined by volume rather than injection speed (14–16). For neuraxial blockade, faster injection speed leads to earlier sensory block onset (17); however, for peripheral nerve block, injection speed does not affect the onset time or duration of sensory blockade (18). Recent studies further clarify the role of speed within the epidural space: higher injection speeds do not produce higher sensory block levels, and lower speeds are associated with a lower incidence of hypotension (19, 20). Additionally, ONSD correlates positively with injection volume (4, 9). Collectively, volume determines total distension (peak ONSD and block extent), whereas speed primarily affects the timing of peak ONSD.

The mechanism linking injection speed to ONSD dynamics remains incompletely understood. The optic nerve is surrounded by a distensible subarachnoid space, and cephalad cerebrospinal fluid (CSF) shift following caudal injection has been proposed (21). Higher injection speed generates a greater peak epidural pressure (22), accelerating CSF cephalad movement and leading to an earlier ONSD peak, whereas slower injection produces a more gradual shift and a later peak. Although epidural pressure returns to baseline rapidly after injection (23), ONSD elevation persisted for at least 40 min in our study. This prolonged elevation likely reflects slow CSF reabsorption and spatial compartmentalisation within the optic nerve sheath: the subarachnoid space is subdivided by arachnoid trabeculae and a mesothelium into interconnected functional compartments (24–26), which create local pressure gradients and delay CSF clearance (25, 26). Notably, no patient developed clinical signs of intracranial hypertension. These findings indicate a dissociation between epidural pressure and ONSD dynamics, explained by the slow CSF reabsorption rate along the optic nerve (0.5–0.8 mm/s) (27) and the trabecular compartmentalizing effect (24–26). Thus, even after epidural pressure normalizes, redistributed CSF remains trapped, sustaining ONSD widening without global hypertension. Given this dissociation and the speed-dependent timing of ONSD elevation, continuous ONSD monitoring is valuable for detecting harmful pressure changes from repeated or continuous administration in prolonged blocks.

Provided adequate clinical effect, we advocate a low-volume regimen for pediatric caudal block. In our 0.5 mL/kg regimen, neither injection speed produced clinical evidence of elevated ICP, and peak ONSD remained below the 5.75 mm threshold (11). Higher volumes have been associated with reduced cerebral blood flow velocity and oxygenation, increased ICP, transient EEG changes, and even ONSD exceeding the threshold (6–8). Using 0.2% ropivacaine at 0.5 mL/kg (a recommended dose), continuous ultrasound visualisation ensured 100% block success and satisfactory anesthesia without opioids, supporting this low volume for perineal procedures (28). Recent work shows that lower dermatomal levels require less than 1 mL/kg, and ultrasound can individualise dosing (16). Building on our real-time guidance that guaranteed success, future research should use ultrasound to explore even more individualized and potentially lower volumes, moving toward personalized caudal block in children.

Both speeds appear safe, but a high speed (1 mL/s) achieves an earlier peak, which is not a clinically desirable outcome. Slower injection has been associated with a lower incidence of hypotension and, by prolonging injection time, may attenuate the impact on cerebral blood flow velocity (9, 19, 20). Therefore, to minimise potential effects on ICP and cerebral hemodynamics, we recommend slower injection, resulting in more gradual ONSD widening and delayed peak, and future studies should directly investigate the speed volume interaction, as slow injection may be more protective when larger volumes are required.

Several limitations should be acknowledged. First, ONSD was measured at only five discrete time points, which precludes precise determination of the true peak time; future studies with higher temporal resolution are warranted. Second, the study used a low injection volume (0.5 mL/kg) and excluded laparoscopic procedures; therefore, the findings may not generalize to higher volumes or settings with elevated intra-abdominal pressure. Third, the sample was limited to male children from a single center, which may limit generalisability to females or other populations. Fourth, we did not employ depth of anesthesia monitoring (e.g., bispectral index or entropy), although all patients received a standardized propofol infusion and ventilation protocol; we cannot completely exclude the possibility that anesthetic depth influenced our ONSD results. Future studies incorporating depth of anesthesia monitoring are warranted to confirm our findings.

## 5 Conclusions

In children undergoing low-volume caudal block for perineal surgery, injection speed modulates the temporal dynamics of ONSD without affecting maximal ONSD, systemic hemodynamics, or EtCO₂. All ONSD values remained below the safety threshold, and no adverse events occurred. Both speeds are safe, but the slower rate (0.1 mL/s) is more suitable for routine clinical practice as it produces a more gradual ONSD rise and minimises intracranial dynamics. However, further research should investigate speed–volume interactions and ultrasound-guided individualisation of dosing. Ultimately, refining procedural details with patient safety as the priority remains key to optimizing pediatric neuraxial block.

## Data Availability

The original contributions presented in the study are included in the article/Supplementary Material, further inquiries can be directed to the corresponding author.
